# Online health communities influence people’s health behaviors in the context of COVID-19

**DOI:** 10.1371/journal.pone.0282368

**Published:** 2023-04-13

**Authors:** Xu Chen

**Affiliations:** Henan Vocational College of Nursing, Wenfeng District, Anyang, China; University of Science and Technology of Fujairah, YEMEN

## Abstract

The online health community has the functions of online consultation, health record management and disease information interaction as an online medical platform. In the context of the pandemic, the existence of online health communities has provided a favorable environment for information acquisition and knowledge sharing among different roles, effectively improving the health of human, and popularizing health knowledge. This paper analyzes the development and importance of domestic online health communities, and sorts out users’ participation behaviors, types of behaviors, and continuous participation behaviors, influence motives, and motivational patterns in online health communities. Taking the operation status of the online health community during the pandemic period as an example, the computer sentiment analysis method was used to obtain seven categories of participation behaviors and the proportion of various behaviors of online health community users, and the conclusion is: the emergence of the pandemic, making the online health community a platform where people are more inclined to choose to consult health issues, and user interaction behaviors have become more active on the platform.

## 1 Introduction

### 1.1 Development status of the online health community

Online health community mainly refers to the online community in which online users describe the disease state according to medical and health-related information in the Internet environment, such as medical knowledge sharing, interaction among health community members, expert consultation and other behaviors. The information in the online health community is mainly about health topics and is highly professional. In addition, the online health community not only includes information acquisition methods such as browsing, searching, and consultation, but also conducts behaviors such as publishing medical information, answering other people’s health questions, and sharing treatment experiences and feelings [1].

Driven by policies such as ‘Healthy China 2030’ and ‘Internet + Healthcare’, online health communities have developed rapidly and are favored by users. At present, there are many forms of applications in the construction of healthy communities in China, among which there are representative online health communities of ‘suffering-suffering type’, such as ‘lung cancer help’, ‘sweet home’, ‘baby tree’, etc. ‘Patient-type’ online health communities, such as ‘Chunyu Doctor’ and ‘Quietly Answered’. These are already online health communities in China.

The online health community, as a key platform for disseminating health information and popularizing medical knowledge, under the background of increasingly mature Internet technology, the concept of ‘Internet + medical health’ was proposed and actively promoted as a national strategy. With the release of the Opinions on Promoting the Development of ‘Internet + Medical Health’ by the General Office of the State Council, various types of online health communities have sprung up in people’s daily lives. In the regular briefing on policies of the State Council on April 16, 2018, experts such as Zeng Yixin, deputy director of the National Health Commission, made policy interpretations on the relevant issues involved in the "Opinions", and the online health community mainly includes the following two aspects:

The first is to improve the "Internet + medical and health" service system. Promote the integration of the Internet and medical and health services in terms of medical treatment, public health, family doctor signing, drug supply guarantee, medical insurance settlement, medical education and popularization, and artificial intelligence applications. Therefore, it covers many aspects of the "three medical linkages" of medical treatment, medicine, and medical insurance.

The second is to improve the support system of "Internet + medical health". Relevant measures were put forward in terms of formulating and improving relevant supporting policies in a timely manner, accelerating the realization of the exchange and sharing of medical and health information, establishing and improving the "Internet + medical and health" standard system, improving the level of hospital management and convenience services, and improving the infrastructure security capabilities of medical institutions.

Due to the support of national policies and attention, the development of online health communities has also been accelerated. More and more users prefer to use this new online platform to understand their own health status, which is more convenient and quicker, independent of time and space. Restricting consultation with experts and doctors, this new era of online diagnosis subtly affects users’ health information needs and behaviors.

Since December 2019, the new crown pandemic has become an inseparable factor affecting the daily life of Chinese people. Terms such as nucleic acid, home isolation, and static blockade have become well-known words. However, for the elderly, as a vulnerable group in society, home isolation brings them more than simply staying at home. Psychologically, the elderly are prone to fear or anxiety; second, their own physical health problems. During the isolation period, the elderly cannot go out to exercise regularly, and a series of physical diseases will occur. Time to get professional medical treatment and missing the best time for recuperation will bring great obstacles to the life and health of the elderly. So this paper focuses on how to use or enhance online health community functions to help the elderly overcome the difficulties brought by the pandemic.

### 1.2 The importance of developing online healthy communities in the context of the pandemic

Creating a healthy community is one of the important tasks of community work, and it is the need to create a harmonious society. Vigorously promote the construction of a healthy community platform, establish a long-term mechanism for health promotion work, give full play to the subjective active role of members, and enhance conscious participation. Provide online medical and health services for the Chinese people during the pandemic, especially to ensure the health and safety of the elderly, and lay the foundation for building healthy cities in China.

The research significance of this topic is embodied in two aspects: theoretical significance and practical significance. In theory, this topic adopts scientific quantitative research on the impact of online health community platforms on participants’ use behavior and patients’ health, solves important issues in the field of medical informatization and online medical research, and helps to establish a more reasonable online medical operation. In practice, the research of this topic can help both doctors and patients to better understand and use online medical care, help doctors follow up and grasp the patient’s condition and psychology, help patients understand their own condition and obtain social support, and have a more positive attitude. face disease. The research on the influencing factors of user behavior in the online medical community can provide directions and suggestions for operators to improve the quality and efficiency of community operation services.

## 2 Literature review

### 2.1 User participation behavior in online health communities

Users of online health communities are often patients or caregivers with certain health problems, including certain health information providers [2]. From the perspective of traditional information science, the behaviors of online health community users mainly include health information browsing behavior, information seeking behavior, information sharing behavior and information utilization behavior. Information on symptoms, etiology, diagnosis, treatment, prevention, and experience [3] conducts a series of activities and makes sense to fulfill their own information needs. Many studies have also found the role of emotion and psychological state in this process, such as the user’s emotional state from uncertainty, confusion, frustration, and doubt to later clarity, confidence, Satisfaction or disappointment, etc. [4]. The above-mentioned emotional or psychological states are mainly based on the analysis and interpretation of the satisfaction of information needs. Some scholars combine the "participation" behavior with online community user research to view a series of behaviors or activities of users in the online health community from a more holistic perspective and conduct certain research on the behavior of users participating in the online health community in different situations. analyze. For example, Wasko and Faraj [5] believed that people’s willingness to share knowledge in online participation (intangible benefits), and the perception of connected community networks (community benefits); Mo and Coulson [6] Through a survey on the participation of HIV online community users, it was found that compared with the users who participated in the posting, the divers in the community were less likely to receive social support and useful information, and at the same time, they were less likely to receive social support and useful information about themselves and others in the community. User relationship satisfaction is also lower. From the perspective of social support, Zhou [7] found that the behaviors of users participating in online health communities mainly include knowledge sharing, emotional support, practical support, network support, respect support and off-topic behavior. However, although the above studies try to look at the behavior of online health community users from a broader sociological perspective, and gradually use the word "participation" to express and study, there is a little connotation of online health community users’ participation behavior. and types are clearly defined or are often confused with information behaviors. Based on this, the author believes that it is necessary to combine the existing research results to make a relatively clear explanation of the user’s participation behavior in the online health community.

The user participation behavior of the online health community refers to the exchange and communication between patients and doctors on medical information and health information in the online health community platform [8]. It is a series of active behaviors in the community such as publishing needs, sharing information, communicating emotions, establishing relationships, etc. Compared with user information behavior, user participation behavior has two differences: first, user information behavior regards users as independent subjects outside the community, and uses online communities to meet their information needs; while user participation behavior regards users as independent subjects outside the community. Part of the community, it emphasizes that the user’s own participation behavior is the constituent element of the community structure and content; the second, the user information behavior is the information search, sharing, dissemination and other behaviors that take the community as an information platform and carry out in it; and user participation Behavior is a complex set of behaviors that take a community as a "social" platform and take place within it. In other words, user engagement behavior includes both information behaviors and social behaviors such as expressing emotions, establishing relationships, and even realizing economic benefits. According to different dimensions, the user’s participation behavior in the online health community can be divided according to the participating objects, the types of social support obtained, and social network behaviors. In a narrow sense, online health community user behavior refers to the exchange and communication between patients and doctors on medical information and health information in the online health community platform [9].

### 2.2 Types of user participation behaviors in online health communities

According to participants, types of social support obtained, social network behaviors, etc., the participation behaviors of online health community users are shown in Table 1. In a broad sense, user behavior refers to a user’s targeted selection of the information he wants to obtain for a certain purpose, and then use the information to determine that this information can act on the subsequent subject behavior. In fact, in this study, user behavior refers specifically to users’ adoption behavior of relevant health information in online health communities.

**Table 1:** Types of user participation behaviors in online health community.

Classification	Category	Meaning
Object	Patient behavior	The patient takes the initiative to realize it, and does not require other roles to participate in it.
	Physician behavior	The doctor takes the initiative to realize it, and does not require other roles to participate in it.
	Doctor-patient interaction	Behaviors that need to be completed by both doctors and patients.
Types of social support	Information support	Information support mainly refers to the transmission and exchange of information between users, including advice, knowledge and relevant treatment experience, etc.
	Emotional support	Emotional support refers to positive emotional expressions that help patients relieve anxiety, such as gratitude, sympathy, encouragement, care, etc.
Online social behavior	Browse	Some users only log in to browse and do not express any opinions, they are called "Divers".
	Comment/Reply Post	A typical online social behavior, including posting and replying.
	Like	Approve the words or actions posted by other users.
	Instant messaging	Send and receive messages instantly.

### 2.3 Continuous participation behavior, influence motivation, and motivation patterns in online health communities

The online health community has grown rapidly in recent years and has become one of the most promising social media services related to health. Current research mainly focuses on the patterns, influencing factors and motivations of continuous participation in online health communities [10, 11]. Some studies have explored the continuous participation of users in online health communities based on behavioral patterns. For example, Massimi M and Liu Mengmeng et al. [12, 13] found that the behavior types of users participating in online health communities are mainly patient-patient interaction and doctor-patient interaction. [14, 15] pointed out that the continuous participation behavior of online health community users can be divided into active participation mode and passive participation mode [16]. Different from the motivation of the initial adoption behavior, the user’s continuous participation behavior may be affected by the factors that appear after the initial adoption [17]. Based on social support theory, some studies attribute the continuous participation of online health community users to Emotional Support, Informational Support and Social Companionship [18, 19]. For example, Wang Y et al. [20] investigated the impact of social support on users’ continuous participation in online health communities, and the results showed that information support had a positive impact on users’ continuous participation behavior. Welbourne J L et al. [21] found through empirical research that emotional support is an important driving factor for users’ continuous participation in online health communities. The study by Zhang S et al. [22, 23] also confirmed that the more information and emotional support users get in an online health community, the lower the risk of interrupting participation. Some scholars have also found that seeking social companionship is the main factor for users to continuously participate in online health communities. Zhang Y [24] showed through qualitative research that social companionship plays a key role in the continuous participation of users in online health communities. Allam A et al. [25] found that social companionship is an important precondition to promoting users’ continuous participation in online health communities. The research of Randall E [26] pointed out that social companionship is a key factor for users to continuously participate in online health communities. In addition, some studies based on motivation theory propose that reputation is the main motivation for users’ continuous participation in online health communities [27]. For example, Wu H et al. [28] found that reputation has a positive impact on users’ continued participation in online health communities. Research by Fiedler M et al. [29] also shows that reputation is an important means to promote users’ continuous participation in online health communities.

To sum up, although the research on the causes of users’ continuous participation in online health communities has formed some theoretical results, the existing research still knows little about the dynamic mechanism that promotes the realization of users’ continuous participation in online health communities, and how to maintain the behavior of online health communities. (Mina, 2020) User awareness of continued participation in online health communities is still limited.

### 2.4 Research questions

In December 2019, COVID-19 broke out in Wuhan, and the pandemic was raging. China and the world have begun a long war against the pandemic. The emergence of the pandemic has seriously affected the daily life of Chinese people, and people’s daily medical treatment and health consultation have been seriously hindered [30]. It is against this background that the online health community began to develop rapidly. What are the specific impacts of online health communities on Chinese health behavior during the pandemic? How do online health communities affect Chinese people’s health behavior? These issues still need to be explored and studied. Based on the basic concepts of the online health community, user adoption behavior and some classic theoretical foundations, combined with the characteristics of the "Lilac Garden" online platform, this paper put forward the following research hypotheses:

**(1) Social support hypothesis**

Social support refers to those behaviors that have a helpful intention. Social support is generally divided into informational support, emotional support, accompanying/accompanying support, tangible support, intangible support, self-esteem support, social network support, materialistic support, and the like. In this study, according to the division of the main behaviors of online health community users, two types of perceived informational support, PIS and perceived emotional support, PES are selected.

H1: Perceived information support significantly affects online users’ health information adoption behavioral intentions.
  **(2) Healthy self-efficacy**

Self-efficacy is an important part of Social cognitive theory, SCT. It refers to people’s confidence in their ability to perform a certain behavior. The higher the individual’s expectation of efficacy, the more inclined they are to make greater efforts. Self-efficacy interacts with information integrity and credibility. The better the individual’s self-efficacy, the better the acquisition and utilization of health information.

H2: Health self-efficacy significantly affects online users’ intention to adopt health information.
  **(3) The moderating effect of homogeneity**

Homogeneity means that people of the same nature are more likely to form social connections. Including gender homogeneity, age homogeneity, regional homogeneity, etc. People tend to have a good impression, communicate and establish connections with people who are similar to themselves, and netizens in the online health community generally have the same or similar purpose, in order to learn about medical and health information. Homogeneity can increase the likelihood of adopting the recommendations in the information through access to correct and useful health information and emotional comfort.

H3: The stronger the homogeneity, the more significant the influence of perceived information support on online users’ health information adoption behavior intention.

## 3 Research design

### 3.1 Research methods

**Fig 1:**
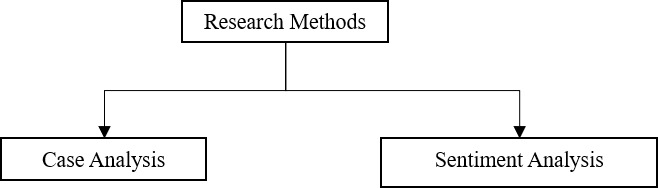
Research methods [13].

The purpose of this study is to investigate the impact of online health communities on the content and types of users’ participation behavior, and to analyze the characteristics of users’ participation in online health communities. It is an exploratory study and is dedicated to discovering new theoretical categories, the flow chart of research methods is shown in Fig 1. The case study method and sentiment analysis method are more suitable for this kind of research, that is, starting from actual cases and problems, through the analysis of the real-world situation reproduction, user participation behavior and presentation attitude, to explore the most essential answers, and try to Find laws and construct theories through cases. This method meets the requirements of academic rigor and can act as an academic bridge, linking the traditional deductive quantitative research paradigm with the qualitative research paradigm [31] Emotion analysis, also known as tendency analysis and opinion mining, is a process of analyzing, processing, concluding and reasoning the subjective texts with emotional colors. Emotion analysis can also be subdivided into emotion polarity analysis, emotion degree analysis, subjective and objective analysis, etc. The purpose of affective polarity analysis is to judge the positive, negative and neutral meaning of the text. In most application scenarios, there are only two types. For example, the words "love" and "hate" belong to different emotional tendencies.

### 3.2 Case study method—Take " Lilac Garden " as an example

**Fig 2:**
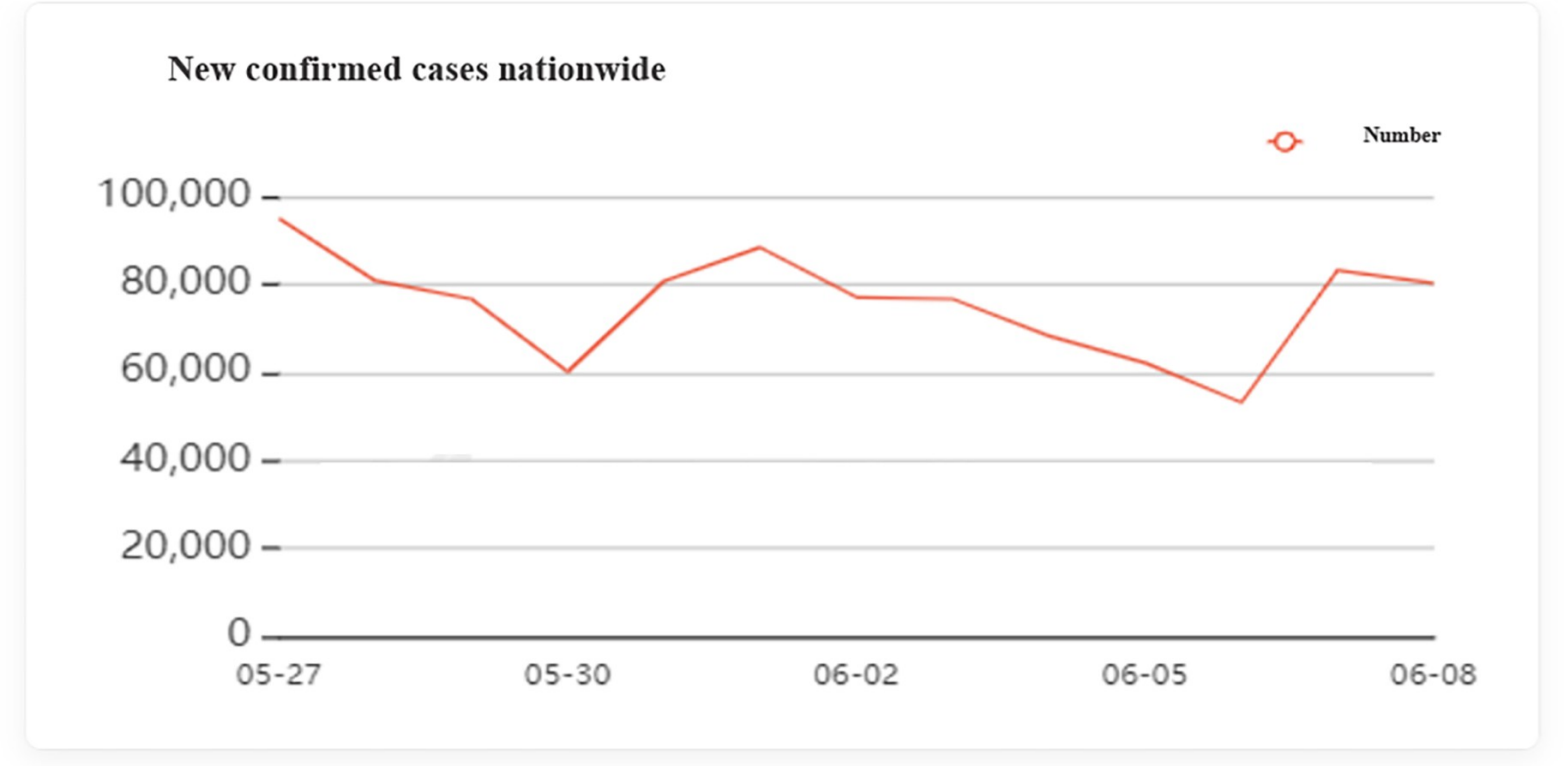
"Lilac Garden" pandemic map real-time broadcast map.

"Lilac Garden" (Fig 2) was founded by Li Tiantian in July 2000, formerly known as "Lilac Garden Medical Literature Retrieval Network". At the beginning of its establishment, it was a social networking site for medical graduate students and medical workers to popularize Internet knowledge, and to use the Internet for medical knowledge retrieval and knowledge sharing. After continuous development, it has become a more authoritative comprehensive forum in the field of biological sciences in China.

The WeChat public account(An application account applied by developers or merchants on WeChat public platform) under "Lilac Garden" has significant media attributes, and the public health science public account represented by "Lilac Doctor" focuses on popular health science. This type of official account has a wide range of users and is the main commercial traffic portal. At the same time, the content of such official accounts plays a greater role in promoting health communication and cultivating health literacy than other types of mass media. Therefore, it is very representative to choose the online health community platform analysis with ‘Dr. Clove’ as an example.

### 3.2.1 The influence of the public account of "Doctor Clove" before and after the pandemic

The emergence of the new crown pandemic has made the online health community platform represented by "Dr. Clove" more and more important, which is intuitively reflected in the number of clicks, readings, and visitors. According to data released by the new media monitoring agency ‘New List’, in 2019, ‘Dr. Clove’ had 290 million cumulative views and 2.75 million cumulative views. There were 3183 articles published in 364 days of the year, of which 1121 claimed originality. The number of reading 10w+ articles reached 2235, and a total of 11093 accumulative appreciations were obtained. The total number of published words in 2019 was 4.925 million words, corresponding to a reading time of 10,260 minutes.

The corresponding period from the end of 2019 to the beginning of 2020 is the initial stage of the pandemic outbreak. According to the data released by the new media testing agency "New List", in 2020, "Dr. Clove" has a cumulative reading of 470 million, and a cumulative reading of 3.95 million. In 365 days of the year, 4,825 articles were published, of which 2,413 were original, and 1,756 of them were about the new crown pandemic. The number of reading 10w+ articles reached 3146, and a total of 59106 accumulative appreciations were obtained. The total number of published words in 2020 is 6.78 million words, corresponding to a reading time of 40,159 minutes.

According to the above content, draw Table 2 as follows:

**Table 2:** Statistics table of "Dr. Clove" WeChat public account before and after the pandemic.

Research variables	Data	
Years	2019	2020
Cumulative readings	290 million	470 million
Cumulative count	2.75 million	3.95 million
Number of articles published	3183 articles	4825 articles
COVID-19 themed articles	126 articles	1756 articles
Read 10w+ articles	2235 articles	3146 articles
Accumulated likes	11093 times	59106 times
Total word count	4.925 million words	6.78 million words
Reading time	10260 minutes	40159 minutes

It can be intuitively seen that the popularity of online health community platforms has increased sharply before and after the pandemic. A large part of the reason is that after the outbreak of the pandemic, the Chinese people’s awareness of their own health prevention has been significantly enhanced. In addition, in 2020, most Chinese people were affected by the time of home isolation, online health communities have become more preferred platforms for Chinese people to browse, and these platforms have also played an important role during the pandemic.

#### 3.2.2 The basic situation and characteristics of "Dr. Lilac" pandemic-related transmission

*3*.*2*.*2*.*1 Pandemic dynamics and professional refutation of rumors*. From January to February 2020, Dr. Lilac has set up a menu entry for "real-time pandemic" specifically for the new crown pandemic, and has five sub-columns including pandemic map, rumor collection, rumor ranking, pandemic subscription and pregnant mothers’ questions and answers.

*3*.*2*.*2*.*2 Pandemic map*. The pandemic map made by ‘ Lilac Garden’ is a real-time pandemic data visualization product. Users can understand the latest progress, real-time broadcast, disease knowledge, etc. of the new coronary pneumonia in real time by viewing the map. From the map, users can intuitively understand the spatial distribution, quantity, and high-incidence areas of the pandemic in real-time, so as to assess the potential risks that may exist in their area. As shown in Fig 2. The figure is taken from the ‘Lilac Garden’ pandemic Forum.

*3*.*2*.*2*.*3 Rumor collection / rumor ranking*. A solicitation column with the theme of "what rumors have you heard about the pandemic" was opened. Users can fill in the message board and submit their own rumors about the pandemic to Dr. Lilac’s team for verification. Once the official account is adopted, the verification results will be displayed in the "Rumour Ranking List". By providing rumors and knowledge, Dr. Lilac makes people soberer to deal with the "battle" of the virus.

*3*.*2*.*2*.*4 Pandemic subscription*. In the early days of the pandemic, the timeliness of data was what people were most concerned about. "Dr. Clove" launched the pandemic dynamic subscription function on January 27, reporting to users the latest situation in the areas they care about in the form of push notifications. On January 30, the new pandemic daily subscription function was launched. The daily Pandemic Daily contains not only a one-day data summary of the areas that users pay attention to, but also a data report written by Dr. Lilac, which interprets the meaning behind the numbers and tries to provide a signal of the development of the pandemic. By February 28, when the "stop update" was announced, the Pandemic Daily has published a total of 30 issues.

*3*.*2*.*2*.*5 Pregnant mother Q&A*. That is, the "Pregnant Mother Community Answers" service. Volunteer doctors from tertiary hospitals provide free one-month community Q&A to pregnant mothers in areas with severe pandemics. The service was later withdrawn.

#### 3.2.3 Content dissemination features

*3*.*2*.*3*.*1 Real-time update*, *comprehensive content*. On January 18, 2020, "Doctor Clove" posted the first article on the new crown pneumonia pandemic, "Interpretation of the latest report of the World Health Organization! 8 Points to Prevent Novel Coronavirus Infection". After January 20, its pandemic-related reports increased significantly. This article focuses on the WeChat push of "Dr. Clove" from January to February. As of February 24, "Dr. Clove" has posted a total of 328 articles, including 195 articles about the pandemic, accounting for about 59.45%. In addition, from the statistical data of release time, the time of release and push is divided into three stages, namely 10:00 in the morning, 12:30–13:30 at noon, and 21:30–22:30 in the evening. Among them, the frequency of publishing pandemic-related news in the morning is less, mainly in the evening, and the number of pandemic-related news is generally 3–4. The release time and the amount of information are in line with the user’s work and rest time and the scope of information reception.

*3*.*2*.*3*.*2 Rich and lively typography forms increase the spread of pandemic prevention information*. The layout of the article is concise and clear, and it is mostly presented in words and long pictures. The article is more readable and avoids the fear of readers reading a lot of text. ‘One Picture Read: ‘The 31 Days Afloat on the Diamond Princess’, ‘Has the New Coronavirus mutated? The World Health Organization’s Q&A is here! mainly uses pictures to display the content, so that readers can get key information at a glance, ‘Simple Rough’, saving readers’ reading time and enhancing the interest of the article.

*3*.*2*.*3*.*3 "Editorial team + medical background" to enhance professionalism and credibility*. The start-up team of "Dr. Clove" has a medical background, and later began to absorb some media-related talents. During the pandemic, "Dr. Clove" invited academicians, CDC researchers, doctors from tertiary hospitals and other people to provide content. "Dr. Clove" also cooperated with the World Health Organization Representative Office in China to launch a collection of protection knowledge.

### 3.3 Sentiment analysis

This article studies the impact of online health communities on Chinese health behavior during the pandemic, and the method that can intuitively reflect people’s behavior in online health communities is to study text, that is, people’s comments in the process of browsing the community, or when consulting their illness. In the process of talking with online doctors, the expression of words is the embodiment of people’s inner emotions. Using sentiment analysis to analyze whether people are negative or positive in the process of browsing the health community can directly judge the online health community for people’s experience is good or bad.

Sentiment analysis in computers is the computational study of people’s opinions, emotions, emotions, evaluations, and attitudes toward products, services, organizations, individuals, problems, events, topics, and their attributes. Text Sentiment Analysis is a common application of natural language processing (NLP), and an interesting basic task, especially for classification to distill the emotional content of the text. It is the process of analyzing, processing, summarizing and reasoning about subjective texts with emotional colors.

In the sentiment analysis method, continue to use "Lilac Garden" as a research case of the online health community. Conduct textual content analysis of this community. It can be seen from the above that the operation time, scale, number of users and content richness of "Lilac Garden" all have the typical characteristics of an online health community, and it can be considered that it has important value and feasibility for data analysis and systematic research.

#### 3.3.1 Data collection

The outbreak of the pandemic started in December 2019. This study extracted the text data of topic posts and reply posts published by community users since the outbreak, mainly from the “Lilac Garden Forum”. The content analysis method is used to analyze the user’s participation behavior type, content and interaction preference in the health community, trying to present an objective text-based user participation behavior pattern in the online health community. Specifically, 2,000 topic posts and 7,529 reply posts were randomly selected from hundreds of thousands of posts in the community by web crawler, with a total of 9,529 posts. Referring to the previous similar research literature [32], more than 9500 random samples were statistically representative and theoretically revealing to a certain extent.

#### 3.3.2 Data analysis

This research is mainly based on the above-mentioned data to carry out qualitative content analysis, that is, to systematically encode, refine and classify the extracted data through the Sentiment Analysis natural language processing model, and discover the theoretical concepts and categories contained in the text [33]. Specifically, this paper attempts to present an overall image of user participation patterns in online health communities with the main line of "participation behavior type-interactive participation preference", which can provide a basic analysis for the subsequent exploration of influencing factors such as user participation behavior in online health communities. Situational and objective interpretive grounds. At the same time, this paper will introduce affective polarity analysis. The sentiment polarity analysis refers to the judgment of positive, negative and neutral texts.

In the ‘Lilac Garden Forum’, the theme posts are usually posted by community operators or an original poster, and the form is usually composed of ‘title + body content’. Since the title is displayed in the list of the main interface of the community, it is the user’s link to the most important entry in the body of the post and is also the main basis for other users to choose to click to enter a post for further reading and participation when browsing the community. Therefore, the title of the topic thread often covers the relative core information recognized by the users who participated in the contribution topic, and to a certain extent reflects the specific type of user participation behavior under the topic line. Based on this, this study starts with the title, and analyzes the specific types of participation behaviors presented by online health community users represented by ‘Lilac Forum’ when they contribute topic posts during the pandemic, and which type of topic posts are more attractive to other users. participate.

#### 3.3.3 Research result

*3*.*3*.*3*.*1 Help-seeking participation behavior*. User participation behaviors for help-seeking mainly refer to online community users who have recognized but not yet clearly expressed their needs, as shown in Table 3. These participation behaviors are often used as the beginning or main verbs with words such as ‘seek for help’, ‘ask’, ‘help me’, ‘excuse me’, and the request objects are usually ‘doctors’, ‘experts’ and ‘kind-hearted people’ ‘experienced people,’ ‘everyone,’ or some of the more prestigious and enthusiastic users in the community sometimes don’t have a specific request verb or object. Through further analysis of the content, the author found that some of the titles only expressed the user’s willingness to ‘seek help’, while another part of the users expressed their willingness to ‘seek help’ on the basis of a brief introduction to the relevant situation, and some users expressed their willingness to ‘seek help’ while ‘seeking help’ Expresses one’s mood or emotional state. However, no specific questions are raised in this type of expression. In this case, non-posting users just look at the title and do not know the clear help needed by users who contributed to the topic thread. Other users can only click on the title to enter the text interface of a single post. to know further.

**Table 3:** Cases of user participation in the “Lilac Forum” during the pandemic.

Serial number	Title	Emotion
7	Help! What should I do if I can’t have my teeth pulled during the pandemic?	Pejorative
11	Help! Help! Don’t dare to go to the hospital during the pandemic, big bosses help	Pejorative
25	[Help] Should I go when I receive a trial call during the pandemic?	Neutral
47	[Help] How to conduct epidemiological investigations in sudden outbreaks?	Neutral
89	We are under the pandemic, I am in Suzhou, where are you?	Neutral
159	I can’t go out to the hospital due to the current pandemic situation, I hope the teachers can help to take a look	Pejorative
273	Help me find out what this is…	Pejorative
…	……	……

It is found that there are emotional or emotional words in a certain number of help-seeking behaviors, and the most expressive emotional state words are "anxious", "fear", "anxious", "urgent", etc.

*3*.*3*.*3*.*2 Questioning participation behavior*. The user participation behavior of questioning refers to the behavior that online community users not only recognize but also clearly express the specific query content. These behaviors often start with words such as "ask for advice", "excuse me", and "consult" as the beginning or main verb, and sometimes there is no specific request verb; the problem expressions contained in the text are usually directed clearly, reflecting the clear needs or goals of the posting users. The questions also have diversified characteristics, such as questions related to the pandemic, such as asking about the harm of new coronary pneumonia, new coronary pneumonia prevention, and new coronary pneumonia government actions, as shown in Table 4.

**Table 4:** Cases of user participation in asking questions in the "Lilac Forum" during the pandemic.

Serial number	Title	Emotion
19	Under the new crown pandemic, how to strengthen the prevention and treatment of osteoporosis in the elderly?	Neutral
36	Remember those classic pneumonia during the pandemic?	Neutral
72	How did the pandemic in Zhengzhou fall?	Pejorative
85	What is the pandemic and what has it brought?	Neutral
141	Can fenugreek cure the new crown and is better than western medicine?	Pejorative
…	……	……

Through combing, it is found that there are occasionally emotional or emotional words in the problem-type participation behavior. The emotional state words that express the most are "anxious", "fear" and "worried", followed by "entangled" and "irritable".

*3*.*3*.*3*.*3 Emotional expression participation behavior*. User participation behavior of emotional expression refers to neither vague help-seeking nor specific questions, nor description of the relevant situation of the user who posted the post, but only the emotion, emotion or psychological state of the post user at that time, such as the confusion of most users about the pandemic, Negative emotional states such as helplessness, worry, timidity, sadness, inferiority complex, fear, fear, hatred, and collapse, as well as positive psychological states such as optimism, calmness, and rationality of some users, and some users hope to gain other people’s opinions in the community. Comfort, blessing, support, etc, as shown in Table 5.

**Table 5:** Cases of emotional expression user participation in the "Lilac Forum" during the pandemic.

Serial number	Title	Emotion
35	Come on Wuhan, come on China	Compliment
78	The neighborhood is closed again, sorry	Pejorative
171	I’m going crazy if the pandemic goes on like this	Pejorative
246	I lost my job during the pandemic, so confused	Pejorative
375	This pandemic has screwed everything up for me	Pejorative
402	I hope the world is free from the pandemic and can be as free as before	Compliment
…	……	……

*3*.*3*.*3*.*4 Experiential descriptive participation behavior*. The user participation behavior of experience description mainly reflects the related experiences of the theme contributors or relatives, such as illness and treatment. Different from the description-type participation behavior, the postings of the experience-descriptive users often do not reflect the need for other users’ problem solving and other reply information support, but mainly describe the individual’s illness, treatment or other related experiences or experiences. The sharing of records and experiences can often bring some reference to other users, as shown in Table 6.

**Table 6:** Cases of user participation behaviors in "Lilac Forum" experienced during the pandemic.

Serial number	Title	Emotion
105	A Wuhan man’s experience in the treatment of new coronary pneumonia in the United States	Neutral
214	A U.S. case infected with the new crown has not recovered for more than ten months. Patient: I can’t get out of bed most of the time	Pejorative
367	A nurse in the United States told the horror story with tears: some of the most serious new crown patients did not actively rescue and were sent to the "deep pit" to die	Pejorative
414	A pharmacist shares his experiences and reflections on suffering from new coronary pneumonia	Neutral
529	After getting the new crown, the sex organs were shortened by 3.8 cm. . .	Pejorative
575	The mental journey of a doctor infected with new coronary pneumonia: When I am well, I have to go to the front line!	Compliment
…	……	……

*3*.*3*.*3*.*5 Participation in knowledge sharing*. Knowledge sharing user participation behavior means that the main purpose of users posting is to share or provide pandemic-related knowledge. After sorting out, it is found that most users share specific knowledge about a certain aspect of the pandemic, such as knowledge released by experts/doctors, knowledge of physical health care, knowledge of treatment and remedies, knowledge of dietary prevention, knowledge of authoritative publications, knowledge of disease inducements, etc.; Experts or experienced people, provide online information consulting services. Such participation behaviors often contain less emotional vocabulary, and more reflect the social support and information-sharing attitudes and behaviors of the posting users, as shown in Table 7.

**Table 7:** Cases of user participation in knowledge sharing of "Lilac Forum" during the pandemic.

Serial number	Title	Emotion
25	The rationality of integrated traditional Chinese and Western medicine in the treatment of new crown	Neutral
62	The Four Most Promising COVID-19 Treatment Options	Neutral
63	New ideas for the treatment of new coronary pneumonia	Neutral
79	Inside the clove circle|my country discovered a new drug for the treatment of the new crown and obtained a patent	Neutral
…	……	……

*3*.*3*.*3*.*6 Social engagement behavior*. The purpose of posting social user participation behaviors is to communicate with more users, such as online greetings, finding or getting to know patients, establishing patient instant messaging groups, etc., as shown in Table 8.

**Table 8:** Cases of social user participation in the "Lilac Forum" during the pandemic.

Serial number	Title	Emotion
140	In the name of psychiatry, find the best you!	Compliment
151	We sincerely invite doctors to join the lumbar spondylolisthesis patient group (there are a large number of cases)	Neutral
173	Do you have any friends in Wuhan?	Neutral
205	Do you have any friends who work in community hospitals in Wuhan?	Neutral
207	Are there any girls who rent a house in Wuhan to prepare for postgraduate entrance exams?	Neutral
…	……	……

*3*.*3*.*3*.*7 Irrelevant engagement*. Irrelevant user participation behavior mainly refers to the content posted by the user that has nothing to do with the online health community and health topics to which they belong. After analysis, it is found that the irrelevant posts posted by users mainly include notifications from online community administrators and posts by community users for the purpose of upgrading their experience points. Behaviors, other advertising behaviors unrelated to the theme of the community, and common holiday blessings, sending and receiving red envelopes and other life-related information posting behaviors, as well as some posting behaviors that have no meaningful content, as shown in Table 9.

**Table 9:** Cases of irrelevant user participation in the "Lilac Forum" during the pandemic.

Serial number	Title	Emotion
111	Posting polls has extra experience, it’s true	Neutral
291	Tomorrow is the new year, I wish you a happy new year in advance	Compliment
292	la la la	Neutral
329	Is it 120?	Neutral
377	Poor spirit	Neutral
…	……	……

The author made basic statistics on the number of the above seven types of participation behaviors, and found that the questioning type of participation behavior was the most (accounting for more than 45%), followed by the help-seeking type of participation behavior (2434:24.6%) and the experience description type of participation behavior (1248:13.1%), and emotional expression. Class participation behavior (334:3.5%), social participation behavior (162:1.7%) and irrelevant class participation behavior (257:2.7%) accounted for the least amount.

### 3.4 Empirical test

#### 3.4.1 Reliability and validity test

In this paper, SPSS24.0 was used to analyze the reliability of the scale. As shown in the test results in Table 10, Cronbach’α coefficient of each latent variable was between 0.90 and 0.95, much higher than the standard of 0.7, indicating that the scale had high reliability. The validity test of the measurement model mainly evaluates the content validity, convergence validity and discrimination validity. The measurement items of each variable in this paper are all from more than 9000 data climbed by the crawler. The analysis of these data ensures the accuracy and comprehensiveness of data sources, so it can be considered that these data have good content validity.

**Table 10:** Reliability test results of sample data.

Latent variable	Measurement item	Standard factor load	Cronbach’α	CR	AVE
R	R1	0.89	0.904	0.908	0.779
	R2	0.93			
	R3	0.81			
A	A1	0.83	0.895	0.896	0.754
	A2	0.90			
	A3	0.86			
T	T1	0.87	0.901	0.902	0.765
	T2	0.83			
	T3	0.91			
S	S1	0.90	0.940	0.938	0.849
	S2	0.95			
	S3	0.90			
FT	FT1	0.88	0.894	0.893	0.728
	FT2	0.85			
	FT3	0.87			
PV	PV1	0.88	0.913	0.893	0.747
	PV2	0.84			
	PV3	0.86			
PU	PU1	0.81	0.905	0.905	0.772
	PU2	0.91			
	PU3	0.90			
SAT	SAT1	0.85	0.900	0.904	0.771
	SAT2	0.91			
	SAT3	0.86			
CU	CU1	0.76	0.908	0.826	0.62
	CU2	0.80			
	CU3	0.79			

To test the convergent validity and discriminative validity of the model, AMOS was used for confirmatory factor analysis. It can be seen from the analysis results in Table 6 that the standard factor loading of all variables is greater than 0.7, indicating that the measurement items can well represent each latent variable. The combined reliability (CR) ranges from 0.826 to 0.938, all higher than the standard of 0.7. The average extraction amount AVE was between 0.62 and 0.849, all of which were higher than the standard of 0.5, indicating that the scale had good convergence validity. The discrimination validity can be tested by comparing the correlation coefficient between the square root of AVE and the latent variable. The calculation results are shown in Table 11. It can be seen that the square root (diagonal) of all latent variables AVE is greater than the correlation coefficient between it and other latent variables, indicating that the difference between measure items is large and the discrimination validity of each latent variable is good.

**Table 11:** Differential validity analysis.

Variable	PU	PV	SAT	CU	FT	S	T	A	R
PU	0.874								
PV	0.785	0.86							
SAT	0.75	0.853	0.874						
CU	0.858	0.793	0.734	0.784					
FT	0.634	0.80	0.689	0.645	0.86				
S	0.475	0.61	0.517	0.484	0.485	0.917			
T	0.585	0.738	0.636	0.595	0.596	0.447	0.87		
A	0.61	0.77	0.663	0.621	0.622	0.466	0.574	0.864	
R	0.65	0.82	0.703	0.661	0.663	0.497	0.611	0.638	0.878

### 3.5 Hypothesis testing

In this paper, AMOS21.0 is used to test the path coefficient of the model and verify the hypothesis relationship between latent variables. The test results are shown in Table 12. It can be seen from the test results that, at the significance level of 0.001, source reliability, information accuracy, timeliness, privacy security and feedback timeliness have a positive influence on the perceived value, among which the influence of information accuracy is the largest, reaching 0.78. Perceived usefulness and perceived value both have a positive impact on user satisfaction. Compared with perceived usefulness, perceived value has a greater impact on user satisfaction. In addition, perceived usefulness has a significant impact on user satisfaction at 0.003 level. User satisfaction has a direct effect on continuous use intention, and the path coefficient reaches 0.927.

**Table 12:** Model validation and hypothesis testing.

Path	Path coefficient	T-value	P-value	Significance	Hypothesis testing
H→PV	0.693	14.22	<0.001	Significance	Establih
A→PV	0.77	14.24	<0.001	Significance	Establih
T→PV	0.738	11.01	<0.001	Significance	Establih
S→PV	0.60	14.93	<0.001	Significance	Establih
FT→PV	0.80	9.39	<0.001	Significance	Establih
SAT→PU	0.191	2.85	0.003	Significance	Establih
CU→SAT	0.927	13.63	<0.001	Significance	Establih

### 3.6 Model fitness test

AMOS21.0 was used to test the fit of the model, and the test results were shown in Table 13. The results show that the absolute fit index, value-added fit index and reduced fit index all exceed the threshold value, which indicates that the research model has good explanatory power.

**Table 13:** Test value of model fitness.

Project	Index	Critical value	Actual value
Absolute fit index	χ2/df	<3	2.137
	GFI	>0.8	0.870
	AGFI	>0.8	0.830
	RMSEA	0.05~0.8	0.061
Value added fit index	IFI	>0.9	0.946
	CFI	>0.9	0.945
	NFI	>0.9	0.91
Reduced fit index	PGFI	>0.5	0.712
	PNFI	>0.5	0.820

In this study, perceived cost consists of three dimensions: source reliability, information accuracy and timeliness. The results show that source reliability, information accuracy and timeliness have significant effects on perceived value, and the path coefficients are 0.693, 0.77 and 0.738, respectively. The results show that the reliability, accuracy and timeliness of information sources in online health communities play an important role in improving perceived value. Online health community is a platform for health information exchange and discussion through Internet media. Due to the lack of face-to-face communication, users can only obtain information through online text. Therefore, the reliability of source and accuracy of information have a great impact on perceived value. Or the health information provided by experienced patients has high reference value. Treatment options and technologies for diseases change over time, and if the information in the community is last edited too long after the time the user searches for it, the value of that information in the user’s mind will be diminished.

Perceived cost consists of two dimensions: privacy security and feedback timeliness. Data results show that privacy security and feedback timeliness have significant effects on perceived cost, with path coefficients of 0.51 and 0.71, respectively. The results show that improving privacy security and feedback timeliness can improve perceived value. The impact of privacy security on perceived value is consistent with previous research results. When users have higher privacy protection, users can obtain the required information with less privacy disclosure, thus improving the perceived value of users in the process of use. When users can get timely feedback on questions and consultations, the waiting time for feedback will be greatly reduced, and users can make timely judgments on disease types to make decisions about the next step. At the same time, timely feedback also enables those diseases affected by treatment time can be effectively controlled, thus improving the user’s perceived value.

In addition, the results show that perceived value has a significant positive effect on user satisfaction, and the improvement of users’ perceived value directly promotes the improvement of users’ satisfaction with online health community. Compared with the perceived value that determines the information quality, the perceived usefulness that determines the system quality has a relatively small impact on user satisfaction, and the path coefficient is only 0.191. This indicates that with the rapid development of information technology, mainstream online health community platforms have basically met the needs of users to obtain health information in terms of providing health services, so that mainstream community platforms of perceived usefulness have basically been realized for users, so the impact of perceived usefulness on satisfaction is relatively small. Finally, user satisfaction has a significant positive impact on continuous use intention, which is the key factor to improve users’ use intention. Managers of community platforms can adopt various means, focusing on improving perceived value to improve user satisfaction, so as to further enhance users’ willingness to continue using, ensure the number of loyal users of online health communities, and promote the sustainable development of online health communities.

## 4 Results and discussion

### 4.1 Online health community

It can be seen from the above results that the initial purpose of users’ participation in online health communities is to meet their health information needs, although this need may be a known and expressed information need for a certain aspect or topic (such as asking questions participation), or an area of health information need that is not clearly recognized (eg, help-seeking participation), or a health information need that may be known but not articulated (eg, descriptive participation). This also confirms the information demand layer model proposed by Taylor [34] to a certain extent. Users usually express their specific level of information needs in an adaptive and nonlinear way. The above three types of user participation behaviors also more reflect the significance of "Lilac Garden" as an online health community to users’ Gap during the pandemic.

### 4.2 Chinese health behavior

On the basis of the above findings, this study also explores what types of topics are more concerned and popular, which also reflects the type of interaction that non-posters prefer to participate in. Under the framework of the text sentiment analysis (Sentiment Analysis) model [26], the responses to various topic posts are also counted, and it is found that the posts during the pandemic are sometimes the first reply to the user who posted the post. Some of these posts are further explanations of the topic posts and supplements. Moreover, it is found that the amount of comments posted during the pandemic is much higher than that before the pandemic, which also shows that the pandemic has made Chinese people more willing to browse and surf on these online health community platforms [35]. Attention has increased significantly, and the development of online health communities has also accelerated significantly with the pandemic.

### 4.3 Hypothesis proof

The results of this study also confirmed the correctness of the article’s hypothesis. First of all, regarding H1, people’s perception of information supports the information adoption behavior of online users. It can be seen from the survey results that people’s information adoption will be based on the surrounding information. During the pandemic outbreak, people’s search frequency for pandemic-related information has greatly increased, and this also confirms the correctness of H3. People with the same or similar purposes can establish closer contacts in understanding relevant information. H2 can also be well confirmed. During the pandemic, people are more hopeful that their lives and health are guaranteed, and they will be more eager to find useful information to fight the pandemic, and the use of health information will be better.

## 5 Research conclusion

This paper uses the text presented by "Lilac Garden" as the basic material to analyze the type of behavior based on user preferences to participate in the interaction. The main contributions are as follows:

(1) This study found that during the pandemic, users’ participation behaviors based on healthy communities mainly include 7 types: asking for help, asking questions, and expressing emotions and so on.
(2) In terms of user active participation, this study found the proportion of user behaviors with different types of problems, reflecting the initial purpose of users participating in the health community during the pandemic to better meet their health information needs during the pandemic.
(3) In terms of user interactive participation, it is found that the amount of feedback obtained by participating behaviors for the purpose of meaning construction is not high, while users of emotional expression and experience description are more likely to attract other users to discuss, reflecting the online health community as a user. The sharing platform has been supported and responded to by the majority of users, but compared with before the pandemic, its role as a knowledge-based and professional information sharing platform has also been greatly improved.

Moreover, it can be seen by comparison that the development of online health communities has a great impact on the health behavior of people, especially during the pandemic, which has given Chinese people great help both physically and psychologically. When encountering problems, more people are willing to seek help from the health community first, and then proceed to the next step after getting a positive answer.

Theoretically, previous studies divided perceived value into social value, interactive value and other dimensions, but paid little attention to the composition of perceived value itself, and rarely analyzed the impact of perceived value on user satisfaction from the perspective of perceived benefits and perceived costs. This study measured perceived value from perceived benefit and perceived cost, and discussed the influencing factors of perceived benefit and perceived cost in online health communities, and proved that the improvement of source reliability, information accuracy and timeliness can effectively improve users’ perceived benefit, and then have a positive impact on perceived value. Higher privacy security and feedback timeliness can improve users’ perceived value by reducing perceived cost. Secondly, by integrating the perceived value evaluation framework with the expectation confirmation model, this study proposed a new influencing factor model of users’ sustainable use in online health communities, which confirmed that perceived value and perceived usefulness affect users’ willingness to continue using through influencing user satisfaction, and enriched the theoretical system of users’ willingness to continue using in online health communities. This study also has certain practical guiding significance for better construction of online health community platform and improving users’ willingness to continue using.

However, there are still some limitations in this study. Although the study case "Lilac Garden" has the typical characteristics of an online health community in terms of users and content scale, it is only an online health community platform and cannot represent all platforms’ laws. Future research also needs to fully consider the differences between different types of healthy communities and extract a more generally applicable logical relationship. Moreover, future research needs to further analyze the multi-dimensional influencing factors and mechanism of user participation in online health communities, so as to better guide the construction of online health communities.
